# Analysis of the nutritional and fatty acid profile of sacha inchi tempe *(Plukenetia volubilis L.)* using different cooking methods

**DOI:** 10.3389/fnut.2025.1527865

**Published:** 2025-02-11

**Authors:** Diana Nur Afifah, Daniaty Afifatus Salam, Fitria Nugraheni, Nina Resti, Rachma Purwanti, Gemala Anjani, Mursid Tri Susilo, Nuryanto, Made Astawan, Irma Sarita Rahmawati

**Affiliations:** ^1^Department of Nutrition Science, Faculty of Medicine, Universitas Diponegoro, Semarang, Central Java, Indonesia; ^2^Laboratory of Sustainable Diets and Biodiversity, Center of Research and Service – Diponegoro University (CORES-DU), Integrated Laboratory, Universitas Diponegoro, Semarang, Central Java, Indonesia; ^3^Doctoral Study Program of Medical Health Science, Universitas Diponegoro, Semarang, Central Java, Indonesia; ^4^Department of Food Science and Technology, Faculty of Agricultural Engineering and Technology, IPB University, Bogor, Indonesia; ^5^Department of Nutrition, Faculty of Medicine, Brawijaya University, Malang, Indonesia

**Keywords:** Tempe, sacha inchi beans, fermentation time, proximate, fatty acids

## Abstract

**Background:**

Indonesia is experiencing food insecurity regarding soybean products. To address this challenge, sacha inchi *(Plukenetia volubilis. L)* has been identified as a potential raw material for making tempe due to the high levels of protein and polyunsaturated fatty acid (PUFA).

**Objective:**

This research aimed to determine the potential of sacha inchi bean tempe on proximate content, PUFA, and the effect of different cooking methods.

**Method:**

Water and ash content were analyzed using the gravimetric method. Moreover, protein, fat, carbohydrates, and fatty acid were evaluated using the Kjeldahl, Soxhlet, difference, and Gas Chromatography (GC) method.

**Results:**

There were significant differences (*p* ≤ 0.05) in water content, ash content, protein, saturated fat, unsaturated, and PUFA in fermentation time. Fermentation increased protein (19.50–20.50%) while reducing water (30.26–28.51%) and PUFA (35.35–32.99%). Cooking methods significantly impacted fatty acids, with steaming retaining the highest PUFA (29.97%) and linolenic acid (14.63%), while frying increased saturated fat (11.24%).

**Conclusion:**

Fermentation process in sacha inchi bean tempe could reduce the water content and saturated fat. This process also increased the ash, protein, and monounsaturated fat content, while the best cooking method was found to be steaming.

## Background

Soybean *(Glycine Max L)* is a type of legume food widely processed and consumed in Indonesia. According to the Central Statistics Agency (BPS), the consumption of this legume food is approximately 3.4–3.6 million tons per capita in 1 year (1). In 2021, Indonesia recorded imports of 2.5 million tons of soybean, which is not sufficient to meet the public’s need for soybeans (1).

Food insecurity regarding soybean raw materials allows for diversification, particularly in processed soybean products, such as tempe. According to the Central Statistics Agency (BPS) in 2021, Indonesia will reach a weekly tempe consumption of 0.146 kg/capita. This value shows that tempe consumption in the country has increased by 4.29% from the previous year (1).

Tempe is a popular Indonesian food with a white, uniform solid and is produced from boiling and fermenting soybean through mold Rhizopus spp. (2). The reaction process of Rhizopus sp. can reduce or eliminate anti-nutritional compounds that are harmful to the body, increase several nutrients such as vitamins, and form beneficial bioactive compounds (3). The making of tempe with long fermentation plays an important role, particularly in the chemical and physical characteristics of tempe (4).

The Amazon tropical forest plant, namely sacha inchi *(Plukenetia volubilis. L)* has recently gained significant attention in the world of food and agriculture. Sacha inchi, known as inka or mountain beans, is a star-shaped plant from the Euphorbiaceae group (5). The bean is widely cultivated in various Asian countries and is often consumed as vegetable oil products. The presence of this plant in Indonesia was first discovered in mid-2018, which continued to increase (5).

Sacha inchi has a good nutritional composition, with a protein content of approximately 22–30%, which is similar to the content in soybean (6). Furthermore, the fat contained at 45–50% has high amounts of polyunsaturated fatty acid (PUFA), with the largest being linolenic acid (35.2–50.8%) and alpha-linolenic acid (33.4–41.0%) (7, 8). Sacha inchi nuts contain EPA and DHA which play a role in preventing cardiovascular disease, protective effects on mood disorders, and cognitive function in children (9). The increasing demand for soybean in Indonesia, along with its import dependence shows the need for diversification in food. The evaluation of different cooking methods is important since PUFA is susceptible to oxidation during heating. Therefore, this research provides valuable insights into how to preserve the nutritional quality of sacha inchi tempe to ensure maximum benefits in dietary applications.

Previous research has been carried out using various plants related to the development of non-soy tempe in Indonesia. Plants that are often used as substitutes for tempe are the Fabaceae (legumes), Gramineas (rice grains), and Amaranthaceae (flowering plants) (10). Several investigations have shown the potential and superiority of developing non-soybean tempe, particularly regarding the nutritional content (10). The development of non-soybean tempe using peanuts as raw material has a protein content of 28.5%, green beans 22.2%, and red beans 33.8% (11, 12). This value is greater when compared with the protein content of soybean tempe, which is 20.8% (13). This rare composition makes sacha inchi a healthier alternative, particularly for addressing cardiovascular health issues.

Cardiovascular disease is the leading cause of global death characterized by disruption of the function of the heart and circulatory system (14, 15). WHO (World Health Organization) reported that cardiovascular disease accounts for 31% of deaths globally (16, 17). Based on the 2018 Basic Health Research (Riskesdas) approximately 1.5, 8.8, and 10.9% of the population experience heart disease, hypertension, and cerebrovascular disease (stroke) (18).

The main factor causing cardiovascular disease is atherosclerosis, namely blockage of blood vessels by plaque due to the buildup of fat and cholesterol. This is mainly influenced by nutritional status, daily consumption patterns, and history of physical activity (19). Modern society tends to live a sedentary lifestyle and consume fast food, which causes health problems such as cardiovascular disease (20). Therefore, there is a need to improve the quality of the diet, such as replacing saturated fat sources with unsaturated types in the form of PUFA and monounsaturated fatty acid (MUFA) (21).

The process of heating food ingredients has several advantages, such as increasing sensory characteristics, nutritional content, digestibility, and absorption of nutrients in the body, alongside reducing the concentration of trypsin inhibitors contained in the ingredients. Frying food can cause damage to some unsaturated fatty acid due to oxidation reactions that occur during cooking (22, 23). Sacha inchi bean tempe is a food ingredient high in PUFA which has the potential to decrease or damage unsaturated fatty acid due to the cooking process (24). The characteristics of unsaturated fatty acid are the presence of double bonds in their hydrocarbon chains and the ability to easily oxidize due to heating. This causes a decrease in hydrocarbon levels during heating (25, 26).

Based on the description, this research aimed to determine the proximate value of sacha inchi bean tempe’s fatty acid and the effect of different cooking methods. The results were expected to provide information to the public regarding fatty acid content of sacha inchi bean tempe in each cooking method. This would allow the public to consider appropriate cooking methods to prevent loss of the unsaturated fatty acid contained in tempe.

## Methods

### Making sacha inchi bean tempe

The making process referred to research on modified sacha inchi bean tempe (27). The raw materials for making tempe consisted of sacha inchi obtained from plantations in the Kuningan Regency area, West Java, and “RAPRIMA” brand yeast containing Rhizopus oligosporus isolate. This process begins with sorting sacha inchi from dirt and foreign objects, followed by soaking for 2 h (28). Subsequently, the selected samples were boiled for 30 min, and washed in the water used for cooking for 24 h or until mucus and foam appeared on the surface of the soaking water. Sacha inchi nuts were washed to remove the epidermis, followed by chopping, boiling for 20 min until cooked, and draining to dryness. The calm and dried sample was sprinkled with tempe yeast amounting to 0.5% of the total weight of the bean and packaged in clear plastic measuring 12×25 cm which was perforated on both sides. The packaged bean was stored in a dark and damp place for 48 h.

### Cooking sacha inchi bean tempe

Cooking sacha inchi bean tempe referred to research related to cooking gembus tempe (29–32). The process started by weighing 25 grams of the sample and cooking according to the specified treatment. Boiling was carried out in water with an initial temperature of 90°C for 12 min, with water: tempe ratio of 2:1 or until completely submerged. Steaming was carried out at an initial temperature of 90°C for 12 min using a steamer pan. Meanwhile, deep frying is conducted by cooking tempe using “Bimoli Special” brand oil at an initial temperature of 130°C for 6 min.

### Proximate analysis

#### Water content

Water content of sacha inchi tempe was analyzed using the gravimetric method by SNI 3144:2015. A sample of 2 g was weighed in a weighing box with the empty weight recorded. Subsequently, the oven was heated to a temperature of 95–100°C and a pressure of ≤100 mmHg (3.94 inch Hg) for 5, hours and cooled with a desiccator. The heating process was measured and repeated for 1 h until a fixed weight was obtained.

#### Ash content

Analysis of ash content in sacha inchi tempe was carried out using the gravimetric method based on SNI 01–2,891-1992 (33). A 2 g of sample was weighed on a porcelain cup with the weight of the cup recorded previously. The ashing process was carried out for the smoke disappeared and ashed in a kiln at a temperature of 550°C for approximately 4 h. This process was carried out until complete ashing, cooled in a desiccator, and measurement was conducted to obtain a constant weight.

#### Proteins

Analysis of protein content in sacha inchi tempe was carried out using the Kjeldahl method by SNI 01–2,891-1992 (33). A 1 g sample was weighed using oil paper or a weighing boat. The sample was transferred to a 300 mL Kjeldahl tube and a mixture of selenium and concentrated H2SO4 was added. The Kjel Digister tool was preheated, followed by storing the 300 mL Kjeldahl tube containing the sample in the tool. Subsequently, the scrubber unit was turned on and destroyed. After 1 h, the KjelDigister tool was stopped, and the tube was removed and allowed to reach room temperature. The tube was attached to the distillation unit, followed by the addition of distilled water and 40% NaOH. The reservoir for the distillation unit installed was 4% H3BO4 in a 250 mL Erlenmeyer. Distillation was carried out until the distillate volume reached at least 3x the volume of the initial container. The distillate was titrated with 0.2 N HCL solution and work on the blank was carried out every digestion cycle.

#### Fat

Fat content in sacha inchi tempe was calculated using the Soxhlet method in accordance with SNI 01–2,891-1992 (33). The process started with weighing a sample of 1 g in a 100 mL beaker. A 25% HCL solution, distilled water, and several boiling stones were added, followed by closing of beaker and placing on the hot plate. The residue was filtered when hot with ash filter paper and washed using hot distilled water. Furthermore, the residue was dried in an oven and placed in filter paper sleeves (hulls). The hulls were put into a Soxhlet apparatus connected to a 300 mL dry fat flask, the weight was recorded and added with hexane. The sample was extracted using hexane solvent. The fat gourd was dried in an oven at 105°C for 30 min or until the hexane aroma was no longer visible. The flask was cooled in a desiccator for 15 min, weighed, and drying stage was repeated to achieve a constant weight.

#### Carbohydrate

Analysis of carbohydrate content was calculated using the by-difference method. During this process, 100% of the nutritional content of the sample was reduced by protein, water, fat, and ash content (34).

#### Fatty acid analysis

Fatty acid analysis was carried out on control samples and the best treatment was decided using the Multi Attribute Decision Making (MADM) method with Simple Additive Weighting (SAW). The results showed that treatment D, namely sacha inchi tempe with 72-h fermentation, was the best treatment in terms of physical appearance (texture, color, and aroma) and organoleptic characteristics (moisture, ash, protein, fat, and carbohydrate content). Analysis of fatty acid levels was carried out using the gas chromatography (GC) method in accordance with SNI 01–2,891-1992.

Fatty acid analysis was carried out using a Perkin Elmer GC, model Clarus 580. The analysis started by extracting fat from tempe as a sample and preparing a standard solution of 1 concentration point in hexane solvent. A 50 mg of sample was weighed and put into a 20 mL screw vial. Aquabides, Methyl tertiary Buthyl Ether (MTBE), and transesterification solution were added, followed by mixing through a vortex for 10 s. The vial was added with hexane and neutralization solution and centrifuged with a balancer until two layers were formed. The organic solution (top layer) was taken using a pipette, placed into a 2 mL vial, and injected using a Gas Chromatography Flame Ionization Detector (GC FID). The groups of fatty acid observed were saturated, MUFA, and PUFA, with types being linoleic (C 18:2, ω6), and linolenic acid (C 18:3, ω3).

## Data analysis

Bivariate analysis in this research used ANOVA and Kruskal-Wallis to determine significant differences between fermentation time and nutritional content, followed by Duncan’s advanced test. The data was also analyzed using an independent t-test to determine significant differences between fermentation process and fatty acid content. Data from research on fatty acid profile of sacha inchi bean tempe including linolenic acid (C 18:3, ω3) were analyzed using the One-Way ANOVA test with the Least Significant Difference (LSD) follow-up test. Meanwhile, data from the analysis of PUFA, linoleic acid (C 18:6, ω6), SFA, and MUFA were analyzed using the Kruskal Wallis test with further tests in the form of the Mann–Whitney test.

## Results

### Physical appearance of sacha inchi bean tempe

**Figure 1:**
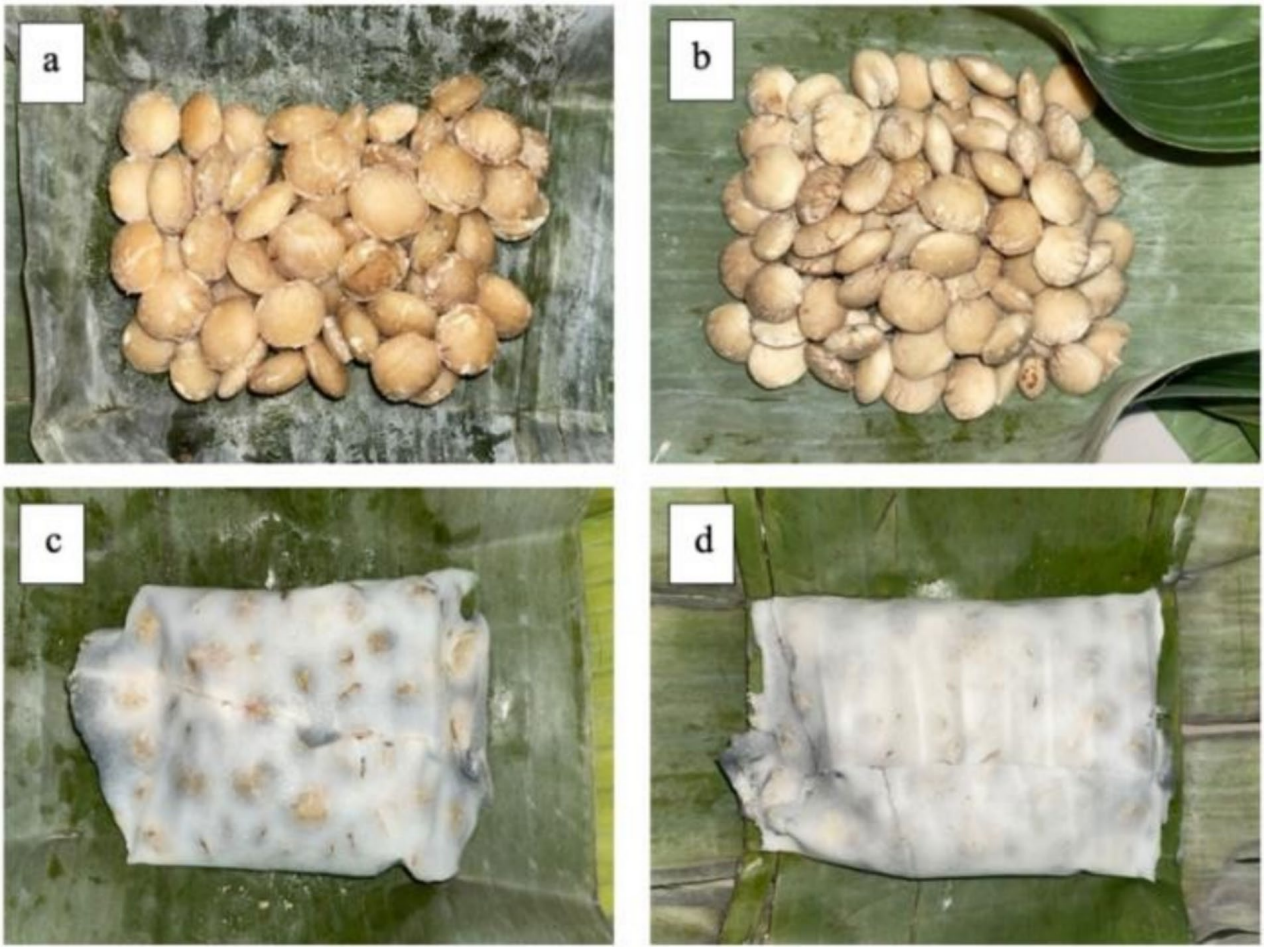
Appearance of sacha inchi bean tempe mycelium growth. **(A)** 0 Hour fermentation; **(B)** 24 Hour fermentation; **(C)** 48 Hour fermentation; **(D)** 72 Hour fermentation.

The growth of mycelium in making tempe using sacha inchi was slower compared to soybean. At 24 h of fermentation, the mycelium started to appear and was still faintly visible (Figures 1A,B). This was similar to the research that showed mycelium growth in koro benguk bean tempe and started to appear after 24-h fermentation (4). The slower growth of mycelium was because of the larger dimensions of sacha inch compared to soybean. These dimensional differences would affect fermentation process, particularly in the penetration of Rhizopus oligosporus mold in making tempe (35). The distinctive aroma of tempe started to be smelled and the mycelium showed rapid growth, which covered sacha inchi at 48 h of fermentation (Figure 1C). This growth continued to thickly cover tempe, which showed grayish white with a very compact texture at 72-h fermentation (Figure 1D). The change in texture, color, and aroma of tempe as fermentation time increased was due to the growth of mycelium. This was caused by protease activity produced through the fungus Rhizopus sp. during fermentation process (4). During the process, the hyphae attached to sacha inchi, causing tempe to have a compact, soft, and white texture (36).

### Nutrient content

Table 1 shows the nutritional content of sacha inchi bean tempe with different fermentation time including water, ash, protein, fat, and carbohydrate content.

**Table 1:** Nutrient content of sacha inchi bean tempe.

Parameter	Fermentation time of sacha inchi bean tempe				*p* Value
	0 Hours	24 Hours	48 Hours	72 Hours	
Water content (%)*	30.26 ± 0.71^a^	29.39 ± 0.89^a,b^	29.28 ± 0.55^b^	28.51 ± 0.73^b^	0.005
Ash content (%)**	1.80 ± 0.02^a^	2.11 ± 0.44^a,b,c^	1.88 ± 0.03^b^	1.97 ± 0.05^c^	0.005
Proteins (%)*	19.50 ± 0.39^a^	19.63 ± 0.39^a^	20.03 ± 0.66^a,b^	20.50 ± 0.29^b^	0.005
Fats (%)**	40.82 ± 0.93^a^	38.03 ± 5.20^a^	38.82 ± 0.80^a^	38.43 ± 3.05^a^	0.201
Carbohydrates (%)**	8.62 ± 1.05^a^	10.85 ± 5.30^a^	10.00 ± 0.43^a^	10.06 ± 3.08^a^	0

*One-Way ANOVA (with Duncan’s follow-up test). **Kruskal-Wallis (with Mann–Whitney follow-up test). The results of superscripts with different symbols (a, b, c) mean there are significant differences between formulations. If the superscripts are the same then there is no difference/same.

### Water content

There was a difference between the average water content and fermentation time in sacha inchi bean tempe (*p* = 0.005). The water content in sacha inchi bean tempe with various fermentation time ranged from 28.51–30.26%. The highest occurred at 0-h fermentation (30.26%) and continued to decrease as fermentation time increased. The water content at 0-h fermentation was significantly different from 48-h and 72-h. Meanwhile, the water content at 24-h fermentation was not significantly different from another fermentation time,

### Ash content

A significant difference was observed between the average ash content and fermentation time in sacha inchi bean tempe (*p* = 0.005). The ash content ranged from 1.80 to 2.11% with the highest obtained in 24-h fermentation (2.11%), while 0-h was significantly different from 48-h and 72-h. The results showed that the value obtained at 24-h fermentation was not significantly different from others.

Protein.

There was a difference between the average protein content and fermentation time in sacha inchi bean tempe (*p* = 0.005). The protein content in sacha inchi bean tempe with fermentation time ranged from 19.50 to 20.50%, with the highest occurring in 72-h fermentation (20.50%). A significant difference was observed at 0-h fermentation (19.50%) compared to 72-h (20.50%). This showed an increase in protein content in sacha inchi bean tempe before and after fermentation.

### Fat

There was no difference between the average fat content and fermentation time in sacha inchi bean tempe (*p* = 0.219). The fat content in sacha inchi bean tempe with fermentation time ranged from 38.03–40.82%, with the highest occurring at 0-h.

### Carbohydrates

There was no difference between the average carbohydrate content and fermentation time in sacha inchi bean tempe (*p* = 0.156). The carbohydrate content in sacha inchi bean tempe with fermentation time ranged from 8.62 to 10.85%, with the highest occurring at 24-h. This was followed by a significant decrease as fermentation time increased.

### Fatty acid content

Table 2 shows fatty acid in sacha inchi bean tempe with fermentation period of 0-h (control) and 72-h (best treatment). There was a significant difference between the average content of saturated fatty acid (SFA), MUFA, PUFA, and linolenic acid of sacha inchi bean tempe after fermentation (*p* < 0.05). However, the average content of linoleic acid showed no significant difference after fermentation (*p* > 0.05). Sacha inchi bean tempe contains 1.6% SFA, 2.68% MUFA, and 32.99% PUFA with linoleic acid of 14.18% and linolenic acid of 18.82%.

**Table 2:** Sacha inchi bean tempe fatty acid.

Parameter	Fermentation time of sacha inchi bean tempe		*p* Value
	0 Hours	72 Hours	
SFA (%)*	2.59 ± 0.11	1.60 ± 0.04	0.000
MUFA (%)*	2.38 ± 0.03	2.68 ± 0.08	0.000
PUFA (%)*	35.35 ± 0.43	32.99 ± 0.65	0.000
Linoleic Acid (%)*	14.30 ± 0.09	14.18 ± 0.36	0.536
Linolenic Acid (%)*	21.06 ± 0.35	18.82 ± 0.38	0.000

*Unpaired T-test.

Table 3 shows fatty acid content of sacha inchi bean tempe in different cooking methods, including PUFA, linolenic acid (C 18:3, ω3), linoleic acid (C 18:6, ω6), SFA, and MUFA. The results showed that the cooking method had a significant effect (*p* = 0.00) on fatty acid profile of sacha inchi bean tempe.

**Table 3:** Fatty acid content of Sacha Inchi bean Tempe.

Parameter	Raw tempe	Boiled tempe	Steamed tempe	Fried tempe	*p* Value
PUFA (%)*	27.70 ± 0.22^b^	27.79 ± 1.55^b^	29.97 ± 0.75^a^	21.25 ± 2.24^c^	0.000
Linolenic Acid (%)**	12.42 ± 0.20^b^	13.73 ± 0.79^a^	14.63 ± 0.48^a^	9.06 ± 1.18^c^	0.000
Linoleic Acid (%)*	15.08 ± 0.03^a^	13.98 ± 0.74^b^	15.26 ± 0.27^a^	12.15 ± 1.03^c^	0.000
SFA (%)*	2.24 ± 0.15^c^	2.35 ± 0.13^c^	2.64 ± 0.08^b^	11.24 ± 1.29^a^	0.000
MUFA (%)*	3.48 ± 0.05^b^	3.03 ± 0.11^c^	3.37 ± 0.09^b^	12.42 ± 1.76^a^	0.000

*Kruskal Wallis test with Mann–Whitney U test. **One-Way ANOVA test with LSD further test. The results of superscripts with different symbols (a, b, c) mean there are significant differences between formulations. If the superscripts are the same then there is no difference/same.

## PUFA

There was a difference between the average PUFA content of sacha inchi bean tempe cooking method (*p* = 0.00). The PUFA content in sacha inchi bean tempe ranged from 21.58–29.97% with the highest occurring in steamed tempe (29.97%) and the lowest in fried tempe (21.58%). In comparison, the PUFA content in raw tempe was significantly different from steamed and fried tempe, but not substantially varied from boiled tempe (*p* = 0.33).

### Linolenic acid

There was a difference between the average linolenic acid (omega-3) content of sacha inchi bean tempe cooking method (*p* = 0.00). The linolenic acid content ranged from 9.07 to 14.63% with the highest and lowest value obtained in steamed (14.63%) and fried tempe (9.07%), respectively. The linolenic acid content in raw tempe was significantly different from all treatments. However, boiled tempe showed no significant difference compared to steamed tempe (*p* = 0.054).

### Linoleic acid

There was a significant effect (*p* = 0.00) between cooking methods on the linoleic acid content of sacha inchi bean tempe. The linoleic acid content ranged from 12.15 to 15.26% with the highest in steamed tempe (15.26%). The results showed that the raw tempe was significantly different from boiled and fried, but not substantially from steamed tempe (*p* = 0.327).

### SFA

There was a difference between the average SFA content of sacha inchi bean tempe cooking methods (*p* = 0.00). SFA content ranged from 2.24 to 11.24%, with the highest obtained in fried tempe (11.24%). In raw tempe, SFA was significantly different from steamed and fried, but not substantially varied from boiled tempe (*p* = 0.109). In comparison, the frying method affected increasing SFA content in sacha inchi bean tempe.

### MUFA

The cooking method had a significant effect on MUFA in sacha inchi bean tempe (p = 0.00). The MUFA content ranged from 3.03 to 12.42% with the highest value obtained in fried tempe (12.42%). The frying method caused an increase in MUFA content in sacha inchi peanut tempe. Based on the results, raw tempe was significantly different from boiled and fried, but not substantially varied from steamed tempe (*p* = 0.07).

## Discussion

### Nutrient content

#### Water content

Water content is defined as the percentage of water in food products and is generally used as a parameter in shelf life. In sacha inchi bean tempe, the water content (28.51–30.26%) met the Indonesian National Standard for soybean tempe (SNI 3144:2015) of <65% (2). The results showed a significant difference (*p* = 0.005) in the average water content of sacha inchi bean tempe against fermentation time. This was shown by a decrease in the water content as fermentation time increased. Similar results were obtained in the research on kara benguk tempe (*Mucuna pruriens*, L), where a decrease was observed as fermentation time increased (37). The decrease was caused by heat during fermentation process and contact with air which caused some of the water to evaporate and used for yeast growth (37).

Generally, water is attributed to yeast metabolism during fermentation. Since the diameter of sacha inchi tends to be large, the metabolism that occurs is not excessively large (37). The use of packaging also affects the water content, which is related to the rate of vapor transmission (38). Furthermore, the application of leaf packaging, such as banana can increase the rate of vapor transmission fluctuatingly. This is because banana leaf has more pores along the length compared to plastic packaging (38).

### Ash content

Ash is an inorganic residue originating from the combustion of organic components, which shows the total minerals in a material or food product (39). In this research, there was a significant difference in the average ash content of sacha inchi bean tempe with fermentation time (*p* = 0.005). The ash content ranged from 1.80 to 2.11%, with the highest obtained at 24-h fermentation. This increase was in line with the decrease in water content in sacha inchi bean tempe. Research in fermentation process can reduce the amount of water contained in a food ingredient causing a significant rise in ash content (39). Generally, there is a corresponding rise in minerals as the ash content in a food ingredient increases (39).

### Protein

The protein content contained in sacha inchi bean tempe with fermentation time ranged from 19.50–20.50%. This value met the Indonesian National Standard for soybean tempe (SNI 3144:2015) of >15% (2). The results showed a significant difference (*p* = 0.005) between the average protein content in sacha inchi bean tempe. Specifically, it was observed that the protein content rose with increasing fermentation time. Previous research also showed that the protein content in kara benguk tempe *(Mucuna pruriens, L)* and lamtoro gung tempe (*Leucaena leucocephala*) increased with fermentation time (37, 40, 41). This increase showed the activity of Rhizopus oligosporus mold that occurred during fermentation process. The metabolism of Rhizopus oligosporus mold produces protease enzymes causing protein to be degraded into simpler components, namely free amino acid (37). Therefore, a longer fermentation process correlated with a greater amount of protein degraded (37).

### Fat

There was no difference in the average fat content of sacha inchi bean tempe with fermentation time (*p* = 0.201). The fat content ranged from 38.03 to 40.82% with the highest occurring at 0-h fermentation. This value met the Indonesian National Standard for soybean tempe (SNI 3144:2015) which was >7%. Fat is a nutritional component that is not easily used directly by microbes for their survival (42). Fermentation process includes enzymatic activity, where Rhizopus oligosporus mold produces the lipase enzyme, which breaks down fat into glycerol and fatty acid (43) as a carbon source for mold (43).

### Carbohydrates

There was no difference in the average carbohydrate content in sacha inchi bean tempe with fermentation time (*p* = 0.156). The carbohydrate content ranged from 8.62 to 10.85%, with 24-h fermentation producing the highest value. However, a significant decrease was observed as fermentation time increased. Previous research also showed that the carbohydrate content in lamtoro gung (*Leucaena leucocephala*) tempe decreased with time increased (40). The decrease occurred because carbohydrates were the main source of nutrients needed by microbes during fermentation process. This occurred due to the enzymatic activity of the Rhizopus oligosporus mold which digested carbohydrates, causing a significant decrease in hexose and slow hydrolysis of stachyose (4).

### Fatty acid content

There was a significant difference between the average content of SFA, MUFA, PUFA, and linolenic acid in sacha inchi bean tempe against fermentation time of 0-h and 72-h (*p* < 0.05). Meanwhile, the average content of linoleic acid showed no significant difference against fermentation time of 0-h and 72-h (*p* > 0.05). There was an increase in the content of MUFA in sacha inchi tempe after fermentation process. A significant decrease was observed in SFA and PUFA including linoleic as well as linolenic acid.

The increase in fatty acid in sacha inchi bean tempe after fermentation was caused by the presence of lipase enzymes produced by Rhizopus oligosporus mold (44). According to Wang et al. during the first 12-24-h fermentation, Rhizopus oligosporus would produce lipase enzymes capable of hydrolyzing fat into fatty acid and will peak at 36-h (45). Subsequently, fatty acid is used by Rhizopus oligosporus for assimilation (45). In this research, the highest fatty acid content in sacha inchi tempe was PUFA of 32.99% with the largest obtained in linolenic (18.82%) and linoleic acid (14.18%).

According to the European Food Safety Authority (EFSA), food product is considered high in PUFA when 45% of fatty acid come from PUFA and produce 20% of the energy. This shows that sacha inchi bean tempe is high in PUFA because 88.52% of fatty acid come from PUFA which meet 63.14% of the product’s energy. The high content of PUFA is due to the chemical characteristics of the raw materials used. Previous research stated that the fat content of sacha inchi bean was 77.5–88.4%, with the largest being linolenic (35.2–50.8%) and alpha-linoleic acid (33.4–41.0%) (7).

Linolenic (C 18:3, ω3) and linoleic (C 18:2, ω6) are essential fatty acid that are beneficial for the body to prevent cardiovascular disease. Based on NHLBI, the recommended PUFA in the DASH diet is 12.6 g per 2000 kcal. Therefore, the recommended amount of sacha inchi bean tempe is 40 g or 2 medium pieces (46). The presentation also needs to be considered, as tempe should be steamed or boiled and not fried to optimize the health benefits and reduce the risk of nutrients lost during the processing process.

Fatty acid is composed of straight hydrocarbon chains with methyl groups (CH3) at one end and carboxyl groups (-COOH) at the other end. This aliphatic monocarboxylic acid is released from triglycerides through hydrolysis, serving as a source of volatile compounds that contribute to the aroma of a food product. Based on their chemical structure, fatty acid is categorized into SFA and unsaturated fatty acid, which consist of PUFA and MUFA (47, 48).

Fatty acid content of raw sacha inchi bean tempe in this research was dominated by PUFA of 27.70%, including linoleic (15.08%) and linolenic (12.42%). Fermentation time used was 48 h, with the type of tempe packaging in the form of plastic. Fermentation time could cause differences in fatty acid levels due to the diversity of lipase enzyme activity from the mold (49). Generally, lipase enzyme plays a role in breaking down fat into linolenic, linoleic, and palmitic acid. Based on previous reports, it was observed that lipase enzyme activity increased rapidly at 36-h fermentation but decreased as fermentation time increased (50).

Fermentation process alters fatty acid profile in food through lipase activity and the interaction of free fatty acid with other compounds. Reactive free fatty acid may form complexes with proteins, carbohydrates, or phenolic compounds, leading to a significant reduction in their levels. Microbial activity during fermentation also produces enzymes that affect the degradation or resynthesis of fatty acid based on several factors such as substrate, pH, temperature, and fermentation duration.

The fluctuation in fatty acid levels during fermentation is influenced by oxidation, hydrolysis, and microbial metabolism. Oxidation can modify the structure of fatty acid, particularly unsaturated, while triglyceride hydrolysis releases free fatty acid and glycerol. Additionally, some microbes may synthesize new fatty acid from available metabolites. These dynamic changes shape the nutritional and functional quality of fermented products.

The results showed that the cooking method had a significant effect on fatty acid content of sacha inchi bean tempe (*p* = 0.00). The highest PUFA content was found in steamed tempe (29.97%) consisting of linoleic (15.26%) and linolenic acid (14.63%). PUFA content in steamed tempe was higher when compared to raw and other cooking methods. However, these results differed when compared to other reports showing that gembus tempe with steaming treatment had lower linolenic and linoleic acid levels than raw tempe (29).

Steaming is a traditional food heating process that depends on steam cooked in a closed container. The higher PUFA content in steamed tempe compared to other treatments can be caused by the steaming process capable of deactivating peroxidase. Therefore, unsaturated fatty acid tends to increase because of the low production of peroxide value. The increase in unsaturated fatty acid levels is also influenced by the loss of water content in food, degradation of organic compounds, and hydrolysis at temperatures of 100–110°C. The steaming process provides an oxygen-free environment capable of inhibiting fat oxidation to reduce the rate of fat damage (51).

Sacha inchi bean tempe with boiling method has lower PUFA, MUFA, and SFA content compared to others. The low content of fatty acid in boiled tempe can be influenced by the occurrence of hydrolysis reactions and the breakdown of fat components into volatile products that are easily evaporated and soluble in boiling water (52).

By using deep frying method, sacha inchi bean tempe shows the lowest amount of PUFA at 21.57%, with higher MUFA and SFA content compared to others. The MUFA and SFA content in fried tempe is 12.46 and 11.24%, respectively. The use of cooking oil as a frying medium can cause an increase in saturated fat due to the absorption of cooking oil into tempe. During the frying process, food ingredients will lose water content due to evaporation which is replaced by cooking oil (25). The cooking oil used in this research has SFA of 37% and MUFA of 42%. Similarly, previous research on tempe gembus with frying treatment showed higher SFA and MUFA compared to other cooking methods (29).

The deep frying method causes low PUFA content, including linolenic and linoleic acid in saca inchi bean tempe. The low PUFA content in fried tempe can be influenced by the oxidative decomposition reaction of unsaturated fatty acid. This shows a condition where the double bonds in PUFA are damaged due to free radicals formed during the frying process. Samples with different treatments showed higher linoleic acid content (C 18: 6, ω6) compared to linolenic acid (C 18: 3, ω3). The low levels of linolenic acid in this research are influenced by the oxidation reaction that occurs during tempe processing. The oxidation reaction begins with the formation of hydroperoxides (ROOH) which are tasteless and colorless, thereby limiting identification through the senses. Lilonenic acid is more easily oxidized due to high content of unsaturated fatty acid. Previous research stated that the presence of more double bonds in food ingredients correlated with a higher rate of oxidation (53). Additionally, linolenic acid was 2.4 times more susceptible to oxidation than linoleic acid and at temperatures of 25–80°C, the amount of hydroperoxide produced was 4 times higher than linoleic acid (54).

Linolenic and linoleic acid can be obtained through food intake because of the inability to be synthesized by the body. This fatty acid plays a role in the formation of healthy cell membranes, regulation of blood vessel blockages, as well as supporting the development and proper function of the nervous system. Their function also includes transport and distribution of cholesterol in the body, along with the lowering of blood triglyceride levels (55). Linolenic acid is a precursor of lipid mediators which have anti-inflammatory properties and play a role in increasing lipoprotein lipase (LPL). These properties inhibit the increase in the breakdown of free fatty acid and glycerol, as well as lipoprotein-triglycerides which is useful in reducing blood triglyceride levels.

Intake of MUFA and PUFA is recommended for healthy individuals and patients with cardiovascular disease. This is because SFA does not have double bonds, which hinders sensitivity to oxidation reactions. Approximately all fatty acid is oxidized in the liver into energy that will be used to activate the function of endocrine glands throughout the body’s tissues and increase cellular combustion. However, excessive intake of SFA can cause fat accumulation because of the inability to oxidize in the liver. There is also a tendency to inhibit the LCAT enzyme (Lechytin cholesterol acetyltrans) and reduce the HDL cholesterol (High Density Lipoprotein) formation factor in the form of Apolipoprotein A-1, thereby causing a decrease in HDL cholesterol levels. The decrease in Apolipoprotein A-1 has an impact on inhibiting the formation of HDL cholesterol. Meanwhile, foods containing SFA can still be consumed with a content of <10% of the total daily energy needed to prevent high LDL levels in the blood (56).

Each cooking method in this research has several advantages and disadvantages. Frying method produces sacha inchi bean tempe with a more savory taste, crispy texture, and attractive yellow-brown color compared to others. However, frying causes an increase in SFA and a decrease in PUFA levels. Steaming method improves sensory characteristics and overall appearance, but plays a role in preventing the loss of PUFA in sacha inchi bean tempe. Therefore, the more recommended cooking method to prevent the loss or damage of PUFA in sacha inchi bean tempe is steaming.

## Conclusion

In conclusion, this research showed that fermentation in sacha inchi bean tempe reduced water and SFA but increased ash, protein, and MUFA effectively. Water, protein, and fat contents obtained met the requirements of SNI. Fatty acid profile, with 32.99% PUFA, comprising 88.52% of total fat, showed a significant potential of sacha inchi as raw material for producing tempe with high PUFA. The results showed that cooking methods significantly affected fatty acid profile of sacha inchi bean tempe (*p* = 0.00). Steaming was the most favorable cooking method, which preserved higher levels of PUFA compared to others. Moreover, deep frying increased SFA levels and decreased PUFA content.

This research only focused on the proximate and fatty acid profile of sacha inchi bean tempe. Therefore, future investigations were recommended on vitamin content and sensory attributes. Limited supporting literature due to novelty research also restricted wider comparative analysis. To address this limitation, further research should be carried out to enable detailed explanations of the nutritional and functional properties of sacha inchi bean tempe.

## Data Availability

The original contributions presented in the study are included in the article/supplementary material, further inquiries can be directed to the corresponding author.
